# End of life content in geriatric textbooks: what is the current situation?

**DOI:** 10.1186/1472-684X-5-5

**Published:** 2006-05-31

**Authors:** Huei Yaw Wu, Farida A Malik, Irene J Higginson

**Affiliations:** 1 Department of Geriatric Medicine, Tan Tock Seng Hospital, 11, Jalan Tan Tock Seng, 308433, Singapore; 2 Department of Palliative Care, Policy & Rehabilitation, King's College London, Weston Education Centre, Cutcombe Road, Denmark Hill, London, SE5 9RJ, UK

## Abstract

**Background:**

Physicians caring for elderly people encounter death and dying more frequently than their colleagues in most other disciplines. Therefore we sought to examine the end-of-life content in popular geriatric textbooks and determine their usefulness in helping geriatricians manage patients at the end of their lives.

**Methods:**

Five popular geriatric textbooks were chosen. Chapters on Alzheimer's disease, stroke, chronic heart failure, chronic obstructive pulmonary disease and lung cancer were examined because of their high mortality rates among the elderly patients. Text relevant to end-of-life care was highlighted. Two reviewers independently coded text into 10 pre specified domains and rated them for the presence of end-of-life information. Content was rated as absent, minimally helpful, or helpful. The proportion of helpful information was calculated.

**Results:**

The textbook with the best end-of-life coverage contained 38% helpful information, the worst had only 15% helpful information. Minimally helpful information ranged from 24% to 50%. As much as 61% of the content in one textbook contained no helpful information at all. Of the ten domains, epidemiology, disease progression and prognostic factors were fairly well covered. Information on advance care planning, ethical issues, decision making and effects of death and dying on patient's family were generally lacking under the individual diseases though they were covered as general topics in other parts of the textbooks. All except one textbook dedicated a chapter to the care of the dying.

**Conclusion:**

This study showed that end-of-life content in geriatric textbooks differed significantly. Most of the textbooks lack good coverage on end-of-life care and more can be done to improve on this.

## Background

Over the past three decades, end-of-life care has become an essential aspect of medical care in many parts of the world. Debates on physician-assisted suicide and euthanasia have further increased interest in palliative care in recent years.

Despite greater awareness amongst medical professionals on death and dying, the results of the Study to Understand Prognoses and Preferences for Outcomes and Risks of Treatment (SUPPORT) [1] in the US showed little evidence of a trend towards improved palliation. Studies in different countries have raised concerns regarding physicians' lack of knowledge on symptom control and national guidelines for such care, as well as poor communication between physicians and patients or their families, are barriers to the provision of good care at the end of life [2-4].

Geriatric Medicine emphasises the holistic care of elderly people. With a rapidly ageing global population, more people are currently dying from chronic diseases as compared to acute illnesses. This trend is projected to continue [5]. Most people who die are elderly. Given that most geriatric patients live out the last stage of life under the care of their doctors, there is an obligation for care-of-the-elderly physicians to be competent in the management of terminally-ill patients. However, improving the end-of-life experience of the dying older persons is complex. Many elderly people still suffer from difficult physical and emotional symptoms in their dying moments [6-8]. As many as one in three conscious elderly patients were found to experience severe pain in the last days of their lives [9].

The American Geriatrics Society's (AGS) position statement on "The care of dying patients" [10] reinforces the need to improve knowledge at all levels of training in end-of-life care amongst physicians and other health care professionals. Although technological advances have enabled easy access to medical information on the internet, textbooks still play an important role in medical education. Three studies conducted during the 1990's examining the end-of-life content in medical textbooks found that coverage was lacking in many disciplines of medicine [11-13]. Geriatric medicine textbooks published in the mid-nineties fared slightly better than other medical textbooks (12). Since then, some publishers and editors have responded positively to the call to improve the end-of-life content in their medical textbooks [14-16]. With so much recent emphasis on quality care for the terminally-ill and dying patients, we wished to examine the latest editions of some of the popular geriatric textbooks published in both the UK and the US, and to determine if the end-of-life content in these textbooks were up to the standard expected of them.

## Methods

### Textbook selection

Five geriatric textbooks: "*Brocklehurst's Textbook of Geriatric Medicine and Gerontology*" (Tallis et al, sixth edition), "*Oxford Textbook of Geriatric Medicine*" (Evans et al, second edition), "*Principles of Geriatric Medicine and Gerontology*" (Hazzard et al, fifth edition), "*The Merck Manual of Geriatrics*" (Beers et al, third edition) and "*Essentials of Clinical Geriatrics*" (Kane et al, fifth edition), were reviewed. The first two were published in the UK, the latter three in the US. All the textbooks reviewed were the latest editions in order to ensure that the most current status of their end-of-life content was being assessed. The UK-published textbooks were chosen based on the results of a local survey conducted to determine the most commonly recommended geriatric medicine textbooks. This survey was carried out by doctors, of a group of physicians specialising in care of the elderly in a South London hospital. The three US-published textbooks were chosen from the list of top ten best-selling geriatric textbooks listed in the *Amazon *website [17].

### Disease and content selection

Five medical conditions were examined. Four of these are predicted to be leading causes of death in 2020- stroke, chronic obstructive pulmonary disease (COPD), lung cancer and congestive heart failure [5]. We added Alzheimer's disease to our review because of its high incidence and prevalence among older people and the emotional and financial burden presented to both the patient's family [18,19] and society. For each disease, we assessed ten domains identified as important in end-of-life care: epidemiology (disease prevalence), disease progression, prognostic factors, symptom management (which encompasses both *symptoms present at the end of life or advanced stage of the disease *and *medical intervention that improves the symptoms of the disease*), mode of death, emotional/social/spiritual issues, advance care planning, ethical issues, decision making, and effect of death and dying on patient's family [see table 1]. Eight of these domains were adapted from an earlier study by Carron et al [11]. Two domains, ethical and emotional/social/spiritual issues, were added because of their importance and relevance to palliative care.

**Table 1:** Textbook coverage of end-of-life care

	**Brocklehurst's Textbook of Geriatric Medicine and Gerontology**	**Oxford Textbook of Geriatric Medicine**	**Principles of Geriatric Medicine and Gerontology-Hazzard et al.**	**The Merck Manual of Geriatrics**	**Essentials of Clinical Geriatrics- Kane**
**Alzheimer's Disease**					
*Epidemiology*	++	++	++	++	++
*Disease progression*	++	+	++	++	+
*Prognostic factors*	0	0	0	++	0
*Symptom management*: *a) Symptom present at eol/advanced stage* *b) Medical intervention that improves symptom*	+ +	+ +	+ +	++ 0	+ +
*Mode of death*	0	0	0	+	0
*Emotional, social and spiritual*	++	+	0	+	++
*Advance care planning*	+	+	0	++	++
*Ethics*	+	0	0	+	+
*Decision making*	+	0	0	+	+
*Effect of death and dying on family*	++	++	+	++	++
**Stroke**					
*Epidemiology*	++	++	+	++	++
*Disease progression*	++	+	++	+	+
*Prognostic factors*	+	++	++	++	0
*Symptom management*: *a) Symptom present at eol/advanced stage* *b) Medical intervention that improves symptom*	+ 0	++ ++	+ 0	++ +	+ 0
*Mode of death*	+	+	+	0	0
*Emotional, social and spiritual*	++	+	0	+	0
*Advance care planning*	0	0	0	++	0
*Ethics*	+	++	0	+	0
*Decision making*	+	+	0	+	0
*Effect of death and dying on family*	++	++	0	0	0
**Chronic Heart Failure**					
*Epidemiology*	++	++	++	+	0
*Disease progression*	+	0	+	+	0
*Prognostic factors*	+	+	++	++	0
*Symptom management*: *a) Symptom present at eol/advanced stage* *b) Medical intervention that improves symptom*	+ +	+ +	+ +	+ +	0 +
*Mode of death*	0	0	+	0	0
*Emotional, social and spiritual*	0	0	+	+	0
*Advance care planning*	0	0	++	+	0
*Ethics*	0	0	++	+	0
*Decision making*	0	0	++	+	0
*Effect of death and dying on family*	0	0	+	0	0
**COPD**					
*Epidemiology*	++	++	++	++	-
*Disease progression*	++	+	++	++	-
*Prognostic factors*	++	++	+	++	-
*Symptom management*: *a) Symptom present at eol/advanced stage* *b) Medical intervention that improves symptom*	++ +	+ +	+ +	++ ++	- -
*Mode of death*	+	+	0	+	-
*Emotional, social and spiritual*	++	+	+	+	-
*Advance care planning*	0	+	0	++	-
*Ethics*	0	+	0	+	-
*Decision making*	0	+	0	++	-
*Effect of death and dying on family*	0	0	0	+	-
**Lung Cancer**					
*Epidemiology*	++	+	++	++	-
*Disease progression*	+	+	+	+	-
*Prognostic factors*	0	++	++	+	-
*Symptom management*: *a) Symptom present at eol/advanced stage* *b) Medical intervention that improves symptom*	+ +	0 +	++ ++	++ ++	- -
*Mode of death*	0	0	0	0	-
*Emotional, social and spiritual*	+	+	+	+	-
*Advance care planning*	0	0	0	+	-
*Ethics*	0	+	0	0	-
*Decision making*	0	++	0	+	-
*Effect of death and dying on family*	0	+	+	+	-

'0'-no information on death and dying; '+' -score 1, includes minimally helpful information on death and dying; '++' -score 2, includes helpful information on care of the dying; '-' -disease not covered in textbook

### Review and rating procedures

Two clinicians, a palliative care physician (FM) and a care-of-the-elderly physician (WHY), independently reviewed the textbooks. For each textbook and each disease, any text mentioning or related to death and dying, the fatal nature of the condition or the course of the disease near death was identified. Text was included for assessment if it was identified by either reviewer, or of course, both.

The reviewers then independently categorised each piece of text according to the domain(s) covered (there could be more than one), and rated it for content. Ratings were '0' (no relevant information, score 0), '+' (minimally helpful information, score 1), and '++' (helpful information, score 2). For example, in the evaluation of the domain on "disease progression", a rating of '+' was given if there was any mention that the disease might lead to death and '++' if each stage of the illness leading to death was being discussed. For "symptom management", '+' was given if the symptoms that might be present at the advanced stage of the disease or the end of life were mentioned anywhere (however briefly) in the text, and '++' if the symptoms present at the end of life were specifically discussed. Any discrepancies or disagreements between the two reviewers in the coding of the domains were documented and referred to a third reviewer (IH).

### Rating consistency

To ensure consistency of the rating process, prior to the assessments, the two reviewers coded and rated a different chapter in one of the five selected textbooks. This process identified and resolved potential differences.

### Analysis

Content scores were available for each domain, for each condition. For the domain on symptom management, ratings for the subcategories were considered separately. The content score for each disease was calculated by summing the scores for the ten domains and expressing it as a ratio over a total score of 22 to allow comparison of the five diseases. The "helpful", "minimally helpful", and "absent" content scores were further analysed and expressed as a percentage across all ten domains. For instance, if the topic on lung cancer in a textbook had two ratings of '++', five of '+' and four of '0', the "helpful" content score is 18.2%, "minimally helpful" content score is 45.5% and "absent" content score is 36.3%.

Content rating ratios were calculated for each textbook, and for individual diseases within these textbooks, using the formula:

Score achievedTotal possible score
 MathType@MTEF@5@5@+=feaafiart1ev1aaatCvAUfKttLearuWrP9MDH5MBPbIqV92AaeXatLxBI9gBaebbnrfifHhDYfgasaacH8akY=wiFfYdH8Gipec8Eeeu0xXdbba9frFj0=OqFfea0dXdd9vqai=hGuQ8kuc9pgc9s8qqaq=dirpe0xb9q8qiLsFr0=vr0=vr0dc8meaabaqaciaacaGaaeqabaqabeGadaaakeaadaWcaaqaaiabbofatjabbogaJjabb+gaVjabbkhaYjabbwgaLjabbccaGiabbggaHjabbogaJjabbIgaOjabbMgaPjabbwgaLjabbAha2jabbwgaLjabbsgaKbqaaiabbsfaujabb+gaVjabbsha0jabbggaHjabbYgaSjabbccaGiabbchaWjabb+gaVjabbohaZjabbohaZjabbMgaPjabbkgaIjabbYgaSjabbwgaLjabbccaGiabbohaZjabbogaJjabb+gaVjabbkhaYjabbwgaLbaaaaa@58D6@

For example, a disease with a content score of 8 out of a possible score of 22 will have a content rating of 0.36. Similarly, a textbook with a total content score of 55 out of a possible score of 110 will be given a rating of 0.5.

## Results

Table 1 shows the coding and ratings of the 10 domains [see table 1], and the appendix shows the chapters assessed [see additional file 1]. The rating process was fairly consistent between the two reviewers, with an inter-rater variability of less than 6%. Ratings can be assessed for both the individual textbooks and the specific diseases [see table 1].

### Analysis by textbook

*The Merck Manual of Geriatrics *has the best coverage on end-of-life care on the whole, with a chapter for every disease and COPD containing the most helpful information across the ten domains studied. *Oxford Textbook of Geriatric Medicine*, *Brocklehurst's Textbook of Geriatric Medicine and Gerontology*, and *Principles of Geriatric Medicine and Gerontology *have relatively similar depth of coverage. Each textbook places different emphasis on different diseases. For instance, *Oxford Textbook of Geriatric Medicine *has good end-of-life content in the chapters on stroke and COPD whereas *Principles of Geriatric Medicine and Gerontology *covers the end-of-life care in chronic heart failure in greater depth. In contrast, *Essentials of Clinical Geriatrics *has little to offer on end-of-life care. It also does not have dedicated chapters on COPD and lung cancer.

The textbooks were ranked according to their content rating from the most to least end-of-life content: *The Merck Manual of Geriatrics *(0.63), *Oxford Textbook of Geriatric Medicine *(0.47), *Brocklehurst's Textbook of Geriatric Medicine and Gerontology *(0.45), *Principles of Geriatric Medicine and Gerontology *(0.45) and *Essentials of Clinical Geriatrics *(0.27) [see table 2].

**Table 2:** Content rating of end-of-life care across the ten domains

**Textbook**	**Disease**	**No information (%)**	**Minimally helpful information (%)**	**Helpful information present (%)**	**Content rating by disease**	**Content rating by textbook**
Brocklehurst's Textbook of Geriatric Medicine and Gerontology (6^th ^edition, 2003)	Alzheimer's Disease	18	45	36	0.59	
	Stroke	18	45	36	0.59	
	Chronic Heart Failure	55	36	9	0.27	0.45
	COPD	36	18	45	0.55	
	Lung Cancer	55	36	9	0.27	

Oxford Textbook of Geriatric Medicine (2^nd ^edition, 2000)	Alzheimer's Disease	36	45	18	0.41	
	Stroke	9	36	55	0.73	
	Chronic Heart Failure	63	27	9	0.23	0.47
	COPD	9	73	18	0.55	
	Lung Cancer	27	55	18	0.45	

Principles of Geriatric Medicine and Gerontology-Hazzard et al (5^th ^edition, 2003)	Alzheimer's Disease	55	27	18	0.32	
	Stroke	55	27	18	0.32	
	Chronic Heart Failure	0	55	45	0.73	0.45
	COPD	45	36	18	0.36	
	Lung Cancer	36	27	36	0.50	

The Merck Manual of Geriatrics (3^rd ^edition, 2000)	Alzheimer's Disease	9	36	55	0.73	
	Stroke	18	45	36	0.59	
	Chronic Heart Failure	18	73	9	0.45	0.63
	COPD	0	36	63	0.82	
	Lung Cancer	18	55	27	0.55	

Essentials of Clinical Geriatrics-Kane (5^th ^edition, 2003)	Alzheimer's Disease	18	45	36	0.59	
	Stroke	73	18	9	0.18	
	Chronic Heart Failure	91	9	0	0.05	0.27
	COPD	-	-	-	-	
	Lung Cancer	-	-	-	-	

### Analysis by disease

*The Merck Manual of Geriatrics *placed fairly equal emphasis on end-of-life content for all the five diseases. Beyond this, contact by disease varied greatly. An end of life content score of greater than 0.5 was achieved in *Brocklehurst's Textbook of Geriatric Medicine and Gerontology *for Alzheimer's disease, stroke and COPD; in the *Oxford Textbook of Geriatric Medicine *for stroke and COPD; in the *Principles of Geriatric Medicine and Gerontology *for chronic heart failure; and in *Essentials of Clinical Geriatrics *for Alzheimer's disease. Chronic heart failure faired fairly poorly in three of the five textbooks, with a score of 0.27 or less [see table 2].

### Analysis by domain

All the textbooks contained a fair amount of information on the domains of epidemiology, disease progression and prognostic factors of the diseases. The domains most lacking in helpful information were "mode of death", "advance care planning", "ethical issues", "decision making" and "effects of death and dying on patient's family" [see table 1]. It was noteworthy that all the textbooks, except for *Essentials of Clinical Geriatrics*, had dedicated a chapter to the care of dying patients [see table 3 and additional file 1]. Symptom management at the end-of-life was well covered in these chapters. Ethical issues, advance care planning, psychosocial issues, spiritual issues, end-of-life decision making and bereavement support were also covered either briefly in the same chapters or in greater detail in other parts of the textbooks. Two good examples were *Principles of Geriatric Medicine and Gerontology *and *The Merck Manual of Geriatrics*, both of which had in-depth coverage on ethical issues and spirituality in the elderly in two separate chapters [see table 3]. It was interesting to find that only *The Merck Manual of Geriatrics *made cross-references to these chapters when discussing end-of-life issues under the individual diseases.

## Discussion

The end-of-life content varied substantially between different geriatric textbooks. Four textbooks contained a fair amount of end-of-life content but less than half of the information available was helpful. Of the five textbooks reviewed, *The Merck Manual of Geriatrics *contained the most end-of-life content. It also dedicated a small section at the end of each chapter on end-of-life issues. This is a reflection of the editorial staff's effort to improve the manual's coverage on end-of-life care following the feedback they received from Carron et al's study [11]. At the other end of the spectrum, *Essentials of Clinical Geriatrics *provided little information that would help the physician care for the dying patient. Information on end-of-life care was hardly available in chronic heart failure and stroke, the most common causes of death in older people. Life-challenging diseases commonly affecting the elderly population like COPD and lung cancer were not covered in this textbook.

Four out of five textbooks dedicated a chapter to the care of the dying patient. However, most of them did not make cross-references to this chapter elsewhere in the text. Including a chapter on palliative care cannot completely make up for the textbooks' lack of end-of-life content under the individual diseases. Most busy clinicians and students who refer to textbooks will probably be selective in their reading. Therefore, the only way for them to learn more about the end-of-life care of a disease is when such information is available in the same chapter or when cross-references to end-of-life content elsewhere in the textbook are made. In our review, *The Merck Manual of Geriatrics *was the only textbook that made cross-references to chapters on care of the dying and ethical issues at the end of life.

Many clinicians associate palliative care with cancer and so might expect textbooks to provide more end-of-life information in the chapters on cancer. This was not supported by our findings. The end-of-life content in lung cancer was generally poor in all the textbooks reviewed. In contrast, the chapters on stroke and COPD contained more end-of-life content in three of the textbooks (*Brocklehurst's Textbook of Geriatric Medicine and Gerontology*, *Oxford Textbook of Geriatric Medicine *and *The Merck Manual of Geriatrics*) while Alzheimer's disease had better coverage on end-of-life care in *Brocklehurst's Textbook of Geriatric Medicine and Gerontology *and *The Merck Manual of Geriatrics *compared to lung cancer. This is encouraging as we now know that non-cancer conditions such as congestive cardiac failure and COPD have the same palliative care needs as cancers [20,21]. By placing more emphasis on end-of-life care in these non-cancer conditions, textbooks can help to highlight the needs of older persons dying from these diseases.

Our study showed that epidemiology of diseases, disease progression and prognostic factors were better covered in the textbooks compared to the other aspects of end-of-life care. Information on advance care planning, decision-making and effect of death and dying on family were often minimally helpful or absent. Even if these issues were discussed, they were usually found in a separate chapter, with no cross-references. Ethical issues at the end of life and bereavement support were usually given insufficient emphasis at the undergraduate level [19] even though they were important measures of quality care at the end of life [20]. Sadly, these areas were often overlooked in clinical practice as well. The SUPPORT study showed that more than half of the patients who died in hospitals were given aggressive treatment in their last days although they would have preferred comfort measures [8]. With advance care planning and end-of-life decision-making receiving more attention in the recent years, it is encouraging to note that many geriatric textbooks have special coverage on these topics.

In their study on end-of-life care content in textbooks from multiple disciplines [12], Rabow et al. reviewed the previous editions of *The Merck Manual of Geriatrics *and *Principles of Geriatric Medicine and Gerontology *(Hazzard et al). As a point of interest, we noted that the end-of-life content in *The Merck Manual of Geriatrics *had improved significantly in the latest edition (third edition). Almost half of the text reviewed in the previous edition (second edition) contained no helpful information on end-of-life care while our results showed that only 13% of the text in the latest edition had no helpful information. *Principles of Geriatric Medicine and Gerontology *also appeared to contain more 'minimally helpful' information and less 'not helpful' information in the fifth edition as compared to the fourth edition. However, we need to be cautious in interpreting and drawing conclusions from these results as the diseases and domains studied were different. Also, the reviewers in the two studies may not have rated the contents similarly.

In the course of our study, we found that *Oxford Textbook of Geriatric Medicine *and *The Merck Manual of Geriatrics *were available electronically on the internet. Clinicians can now have easy access to these textbooks and will be able to refer to them in the wards when queries arise in the middle of their ward rounds, provided computer terminals are available. We hope that the publishers of the other geriatric textbooks will also consider making theirs available online as this will certainly facilitate learning among clinicians and medical students.

This study was limited in that the domain ratings assigned may be unreliable. We tried to overcome this by setting clear definitions for each domain and agreeing on how each piece of text should be rated in the respective domains. This study was also limited by the subjective nature of the methodology. However, with any assessment of medical textbooks, the methodology will nearly always be subjective. Indeed when using textbooks to guide clinical practice, healthcare professionals read the content subjectively. In order to improve inter-rater consistency, we carried out an exercise to improve reliability prior to the actual text review. However, as the two reviewers came from different backgrounds and training, it was interesting to note how the two reviewers viewed some aspects of end-of-life care. For instance, the palliative care physician felt that discussion of the "multidisciplinary approach" of management at the end of life in the text could be considered in the end-of-life content. The care of the elderly physician thought otherwise as he felt that this approach was widely adopted in geriatric medicine and not specific to palliative care. Another example was in the rating of the domains. The care of the elderly physician was less stringent in the rating of symptom management as compared to the palliative care physician who expected more in-depth discussion in this area. In spite of these initial differences in opinion, there was only 6% discrepancy, which was resolved by the third reviewer. Having two reviewers from different medical disciplines was a benefit to this study, as it provided different perspectives and helped to reduce potential blind spots that would have otherwise gone undetected.

Future research might include similar studies assessing current end of life content in major journals and how this has changed over recent years, or assessing the end of life content in textbooks and journals since the inception of educational resource sites online such as the EPEC project [24] (Education in Palliative and End of Life Care) and EPERC (End of Life/Palliative Education Resource Centre) [25]. It might have been interesting to interview textbook editors or chapter authors to understand the differences in the end of life content of their text books; however this would not have necessarily been representable or generalisable to the population of health care professionals who would read the textbooks and would also be subjective.

## Conclusion

The end-of-life content differed significantly in different geriatric textbooks. Important aspects of end-of-life care such as advance care planning, decision-making and effects of death and dying on the family/carers were not given enough emphasis under the individual diseases. Four out of five textbooks provided specific chapters on care of the dying and end-of-life ethical issues, though cross-references were usually not made to these chapters. It was noteworthy that non-cancer conditions received similar, if not better end-of-life coverage, as cancer in geriatric textbooks. It is hoped that the results of this study will encourage the editors and publishers to improve on the end-of-life content in their textbooks, and in so doing, indirectly influence the quality of the care older persons are going receive in the last days of their lives.

## Competing interests

The author(s) declare that they have no competing interests.

## Authors' contributions

WHY participated in design of the study, the rating of the contents of the textbooks, interpreting the results and preparing the manuscript. FAM participated in the rating of the contents of the textbooks, reading, amending and approving the manuscript, corresponding with the editor and answering the reviewers' queries. IJH participated in the design of the study, was the third independent rater when there were conflicting opinions regarding the EOL content rating, participated in reading, amending and approving the manuscript. All authors read and approved the final manuscript.

**Table 3:** Chapters dedicated to end-of-life care or to issues related to the end of life

**Textbook**	**Chapter**	**End of Life Issues Covered**
Brocklehurst's Textbook of Geriatric Medicine and Gerontology (6^th ^Edition)	1)Palliative Care 2)Ethical Issues in Geriatric Medicine	-Symptom management -Advance care planning -Decision making
Oxford Textbook of Geriatric Medicine (2^nd ^Edition)	1)Symptom Management and Palliative Care 2) Legal and Ethical Issues in Geriatric Medicine	-Symptom management -Ethical issues -Advance care planning -Psychological and spiritual issues -Bereavement support -Advance care planning -Decision making
Principles of Geriatric Medicine and Gerontology (5^th ^Edition)	1)Care of the Dying Patient 2)Ethical issues in Geriatric Medicine 3)Spirituality and the Elderly	-Symptom management -Decision making -Bereavement support -Decision making -Advance care planning -Withdrawing and withholding treatment -Euthanasia and assisted suicide -Spiritual issues
The Merck Manual of Geriatrics (3^rd ^Edition)	1)Care of the Dying Patient 2)Legal and ethical issues 3)Social issues	-Symptom management -Emotional, social and spiritual issues -Ethical issues -Advance care planning -Bereavement support -Advance directives -Decision making -Euthanasia and assisted suicide -Spiritual issues
Essentials of Clinical Geriatrics (5^th ^Edition)	1)Terminal Care* 2)Ethical issues in the care of older persons	-Psychological issues -Advance directives -Decision making

* a section within the chapter "Developing Clinical Expectations"

## Pre-publication history

The pre-publication history for this paper can be accessed here:



## Supplementary Material

Additional File 1 1. Appendix : An overview of the chapters covered in the study. The appendix provides an overview of the chapters covered in the study. Click here for file

## References

[B1] The SUPPORT Principal Investigators (1995). A controlled trial to improve care for seriously ill hospitalised patients. JAMA.

[B2] Foley KM (1995). Pain, physician-assisted suicide and euthanasia. Pain Forum.

[B3] Callahan D (1995). Once again reality: now where do we go. Hastings Cent Rep.

[B4] Solomon MZ, O'Donnell L, Jennings B, Guilfoy V, Wolf SM, Nolan K, Jackson R, Koch-Weser K, Donnelley S (1993). Decisions near the end of life: professional views on life-sustaining treatments. Am J Public Health.

[B5] Murray CJL, Lopez AD (1997). Alternative projections of mortality and disability by causes 1990–2020. Lancet.

[B6] Hanson LC, Danis M, Garrett J (1997). What is wrong with eol care? Opinions of bereaved family members. JAGS.

[B7] Desbiens NA, Wu AW (2000). Pain and suffering in seriously ill hospitalised patients. JAGS.

[B8] Lynn J, Teno JM, Phillips RS, Wu AW, Desbiens NA, Harrold J, Claessens MT, Wenger N, Kreling B, Connors AF (1997). Perceptions by family members of the dying experience of older and seriously ill patients. Annals of Int Med.

[B9] Somogyi-Zalud E, Zhong Z, Lynn J, Hamel MB (2000). Elderly persons' last six months of life: Findings from the hospitalised elderly longitudinal project. JAGS.

[B10] American Geriatrics Society Ethics Committee (1995). The care of dying patients: a position statement from the American Geriatrics Society (AGS). J Am Geriatr Soc.

[B11] Carron AT, Lynn J, Keaney P (1999). End-of-life care in medical textbooks. Annals of Int Med.

[B12] Rabow MW, Hardie GE, Fair JM, McPhee SJ (2000). End-of-life care content in 50 textbooks from multiple specialties. JAMA.

[B13] Rabow MW, Fair JM, Hardie GE, McPhee SJ (2000). An evaluation of the end-of-life care content in leading neurology textbooks. Neurology.

[B14] Rabow MW, McPhee SJ (2002). Deficiencies in end-of-life care content in medical textbook. JAGS.

[B15] Rabow MW, McPhee SJ (2001). End-of-life literature. CHEST.

[B16] Rabow MW, McPhee SJ (2001). End-of-life content in textbooks. Am J Psychiatry.

[B17] http://www.amazon.com/exec/obidos/search-handle-form/103-0261074-8265472.

[B18] Haley WE, Levine EG, Brown SL, Berry JW, Hughes GH (1987). Psychological, social and health consequences of caring for a relative with senile dementia. J Am Geriatr Soc.

[B19] Ory MG, Hoffman RR, Yee JL, Tennstedt S, Schulz R (1999). Prevalence and impact of caregiving: a detailed comparison between dementia and non-dementia caregivers. Gerontologist.

[B20] Addington-Hall JM, Lay M, McCarthy M (1996). Dying from heart disease. JR Coll Phys Lomdon.

[B21] Shee C, Ford G, Lewin (1996). Managing late stage respiratory disease. Managing terminal illness.

[B22] Billings JA, Block S (1997). Palliative care in undergraduate medical education. Status report and future directions. JAMA.

[B23] Lynn J (1997). Measuring quality of care at the end of life: a statement of principles. JAGS.

[B24] The EPEC project-Education in palliative and end of life care. http://www.epec.net/EPEC/webpages/index.cfm.

[B25] EPERC: End of Life/Palliative Education Resource Centre. http://www.eperc.mcw.edu/.

