# Supplementary material for: End of life content in geriatric textbooks: what is the current situation?
Source: BMC Palliat Care. 2006 May 31;5:5. doi: 10.1186/1472-684X-5-5 (PMC1501002; doi:10.1186/1472-684X-5-5)
Supplement: Additional File 1 — 1. Appendix : An overview of the chapters covered in the study. The appendix provides an overview of the chapters covered in the study. [file 1472-684X-5-5-S1.doc]

**appppppendix: An overview of the chapters covered in the study**

| **Textbook/ Edition (year)** | **Topic** | **Pages** |
| --- | --- | --- |
| Brocklehurst’s Textbook of Geriatric Medicine and Gerontology/ 6^th^ edition (2003) | Chronic heart failure | 371-384 |
|  | COPD | 495-508 |
|  | Dementia | 775-836 |
|  | Lung Cancer | 509-530 |
|  | Stroke | 587-638 |
|  | Others:  a)Palliative Care  b)Ethical Issues in Geriatric Medicine | 257-272  273-283 |
| Oxford Textbook of Geriatric Medicine/ 2^nd^ edition (2000) | Chronic heart failure | 417-427 |
|  | COPD | 508-521 |
|  | Dementia | 922-931; 1090-1096 |
|  | Lung Cancer | 446 |
|  | Stroke | 451-482; 1082-1090 |
|  | Others:  a)Symptom management and palliative care  b)Legal and Ethical Issues in Geriatric Medicine | 1113-1125  1051-1060 |
| Principles of Geriatric Medicine and Gerontology/ 5^th^ edition (2003)  *-Hazzard et al* | Chronic heart failure | 453-474 |
|  | COPD | 517-534 |
|  | Dementia | 1391-1400; 1479-1486 |
|  | Lung Cancer | 703-714 |
|  | Stroke | 1373-1390 |
|  | Others:  a)Care of the Dying Patient  b)Spirituality and the Elderly  c)Ethical issues in Geriatric Medicine | 323-334  347-351  353-360 |
| Essentials of Clinical Geriatrics/ 5^th^ edition (2003)  *-Kane* | Chronic heart failure | 300-301 |
|  | COPD | - |
|  | Dementia | 129-143 |
|  | Lung Cancer | - |
|  | Stroke | 289-294 |
|  | Others:  a)Terminal care  b)Ethical issues in the care of older persons: Advance directives and EOL Care | 89-90  459-472 |
| The Merck Manual of Geriatrics/ 3^rd^ edition (2000) | Chronic heart failure | 900-914 |
|  | COPD | 779-786 |
|  | Dementia | 357-379 |
|  | Lung Cancer | 800-805 |
|  | Stroke | 397-422 |
|  | Others:  a)Care of the dying patient  b)Legal and ethical issues  c)Social issues | 115-126  127-141  142-155 |
